# Adapted remote cognitive behavioural therapy for comfort eating with a woman with intellectual disabilities: Case report

**DOI:** 10.1186/s40337-022-00537-6

**Published:** 2022-02-22

**Authors:** Chris Millar, Beth Greenhill

**Affiliations:** University of Liverpool, Liverpool, United Kingdom

**Keywords:** Comfort eating, Intellectual disabilities, Remote working, Clinical psychology, CBT, Down syndrome

## Abstract

**Background:**

Diagnostic overshadowing can prevent the treatment of comfort eating in people with intellectual disabilities, and the published literature contains few therapeutic examples. This case study reports a relatively novel, promising, and accessible, remote cognitive behavioural intervention.

**Case presentation:**

This case study documents a therapeutic intervention for comfort eating with a client, Sarah, in a National Health Service adult Community Learning Disabilities Service. Sarah is a white, British woman in her late thirties, with a diagnosis of Down syndrome who experienced significant problems with comfort eating and subsequent weight management. Despite dieting and exercising, Sarah was clinically obese and experienced weight related pain and psychological distress. Systemic intervention between Sarah, her mother, and the therapist formulated Sarah’s eating difficulties using a cognitive behavioural framework. This hypothesised how comfort-eating met her emotional needs and maintained her health difficulties. Remote cognitive behavioural therapy interventions included collaborative behavioural experiments, coping strategies, and homework tasks.

**Conclusions:**

The Maslow Assessment of Needs Scale-Learning Disabilities, Glasgow Depression Scale for people with a Learning Disability, Glasgow Anxiety Scale for people with an Intellectual Disability, qualitative feedback from family, as well as frequency data showed significant improvement. Additionally, the case considers the evidence base, assessment, formulation and intervention, before reflecting on its various strengths and limitations. It reflects on the intersectionality of sexuality and intellectual disabilities, and the desire for romantic attachment, which was additionally complicated by the context of coronavirus and social isolation. The environmental influences on comfort eating regarding this case, and in general, the experiences of people with intellectual disabilities are also considered. The potential clinical impact of this case study includes exemplifying an effective comfort eating therapeutic intervention in an often overlooked client group.

## Background: literature review

The term ‘learning disability’ is a social construction but can be defined as someone who has a ‘significant impairment of intellectual ability’ (IQ less than 70), with ‘difficulties in social and adaptive functioning’ since childhood [76]. This case study uses the term ‘intellectual disability’ (ID) and people with intellectual disabilities (PWID), which denotes a broad inclusion criteria [36], and is increasingly used in UK policy, practice, and research [18].

### Inequalities

PWID face a myriad of societal inequalities [15]. Within healthcare, this includes the over-medication of psychotropic drugs with inadequate review [11], diagnostic overshadowing, such as attributing a problem or symptom to a person's disability [51], inferior care [72], and discrimination from services [25]. These factors may contribute to PWID having a median age of death that is twenty-five years younger than the general population [28].

In regards to mental health, PWID are more likely to receive clinical diagnoses of ‘mental ill-health’ than the general population, with the most prevalent being ‘problem behaviours’. This is especially true for people who have experienced more life events, have less support, and are female [19]. Furthermore, PWID face a greater risk of experiencing stress than those without a disability [31], yet they are likely to have fewer resources available to help them cope [47, 63]. Baum and Lynggaard [5] argue that the systemic discrimination of PWID in the UK and their resultant health disparities may be the result of decades of prior overlooking, underfunding, and service closure. Despite repeated calls for health policy and service improvements from academics [2, 3] and leading charities [52] there has been little progress.

### COVID-19

This lack of progress appears to be highlighted by the unfolding coronavirus (COVID-19) pandemic. This has disproportionately affected PWID who face increased risk of morbidity and mortality [40]. Additionally, human rights concerns were raised with the detrimental impact on quality of life caused by disruptions to normal life [17, 41].

It is critically important that the right to health applies to everyone, including PWID, who have been largely neglected [34]. This is protected by the Human Rights Act [33], and the United Nations Committee on Economic, Social and Cultural Rights [68]. The intersecting health needs of PWID require further research.

### PWID and comfort-eating

Despite evidence of PWID using comfort-eating as a way to destress [16], and the understanding of this as a  psychiatric difficulty within the ‘Diagnostic Criteria for Psychiatric Disorders for Use with Adults with Learning Disabilities (DC-LD)’ [29], little further information exists. Moreover, there are no known psychotherapeutic resources, manuals, guides, or case studies.

### Psychotherapy for PWID

Kroese et al. [39] report PWID have more probability of experiencing ‘cognitive deficits’, such as problem-solving, memory and language. Despite this, these factors are not barriers as therapists can effectively adapt their delivery [73]. These adaptions have led to a range of established approaches including psychodynamic [6], cognitive analytic [7], and cognitive behavioural [35]. Whichever tailored psychotherapeutic modality, Abbott and Howarth [1] list fifty recommendations for supporting PWID, emphasising the importance of person-centred approaches. They argue that some clients’ emotional and sexual needs may be of equal or greater importance than their employment or health needs. Practitioners should strive to understand clients’ unique perspectives,for instance, enquiring the preferred approach when exploring presenting problems that include sexuality with sexual-minority clients [14]. Nevertheless, despite the many adapted approaches, Vereenooghe and Langdon’s [70] meta-analysis found limited efficacy. Similarly, Koslowski et al. [38] found ‘no compelling evidence’ to support mental health interventions for people with mild to moderate ID, however, they noted that cognitive behavioural therapy (CBT) warrants further investigation.

### Cognitive behavioural therapy (CBT)

Beck’s model of CBT [8] states that therapists’ role is in helping clients elicit and modify their beliefs about ‘activating events’ in order to change their emotional and behavioural consequences. This approach is guided by case conceptualisation [42]: a collaborative hypothesis between the therapist and client which synthesises presenting difficulties. Using specific CBT techniques, the client is supported to identify links between their thoughts, behaviours and feelings. This informs interventions to better manage situations, consider alternative explanations, and ultimately reduce distress.

Specific CBT approaches are recommended based on clients’ presenting difficulties. For example, enhanced cognitive behavioural therapy (CBT-E) interventions are used to target negative appraisals of self-worth based on body image and weight [27].

Alternatively, generic maintenance cycles are common cognitive and behavioural principles that can be applied across the spectrum of psychological difficulties. Cycles are idiosyncratic, based on clients’ personal expectations, self-evaluations, rules, and experiences [10].

CBT can be adapted for PWID, and is most efficacious when delivered via individual interventions [70].

### Cognitive behavioural therapy with PWID

Evidence suggests that adapted CBT can help PIWD manage anxiety [45], bereavement [46], depression [69], and a range of other presenting difficulties [32].

Lindsay [44] argues that fundamental aspects of CBT including agenda setting, testing cognitions and homework tasks can be modified to good effect. This includes third-wave dialectical behaviour and mindfulness/acceptance practices [57]. These adaptions and improvements have led to National Institute for Health and Care Excellence (NICE) guidelines recommending adapted CBT for PWID in the prevention, assessment and management of mental health difficulties [55].

## Case presentation: assessment

A referral was made to the psychology team from Sarah’s Community Learning Disabilities Nurse (CLDN), who was providing bereavement support following the loss of her grandfather. The referral stated that Sarah was a woman in her late thirties who was ‘finding it difficult to manage her emotions’, with ‘episodes of comfort-eating’. Sarah had also handed day centre staff leaflets on suicide which she had collected from the GP. This caused concern amongst Sarah’s care team and family.

Information was gathered from discussion with Sarah and the referrer. Supporting information was assimilated from clinical notes and Sarah’s mother Gill, all of which Sarah consented to. Standardised psychometric measures and frequency data were used to assess the presenting difficulties. Due to the coronavirus disease (COVID-19) global health pandemic, the assessment and intervention took place remotely using telephone and video.

The British Psychological Society (BPS) [13] note that ‘ID’ is a social construction signifying an array of challenges likely to be experienced by an individual. This description should exist alongside other relevant descriptions such as ‘family member’ or ‘friend’. Consequently, every effort was made to assess Sarah’s difficulties from her perspective, given her unique personality, interests, experiences, spiritual beliefs, support network, abilities, and sexual orientation.

### Background information

At the time of referral, Sarah was living in supported accommodation with one other resident. Here, she was highly independent, managing her own self-care, attending various clubs, and volunteering at a local homeless charity.

Whilst on the waiting list, Sarah was required to ‘shield’ as people with Down syndrome have an increased risk of death from COVID-19 [22]. Sarah returned to her family home to live with her mother, Gill, and minimised all social interaction to prevent the risk of developing serious medical complications [59].

Sarah had one older sister, Bryony, who had a young family. Sarah felt close with Bryony and described her as her ‘best friend’. Due to COVID-19 social distancing rules, she was only able to see her sister outside from a distance. However, they spoke regularly on the phone.

Sarah spoke fondly of her maternal grandparents, whom she called ‘angels’. Sarah found it upsetting communicating with them through the window, due to COVID-19. She longed to hug her grandparents and sister, having found the lack of physical touch distressing.

Whilst living in supported accommodation, Sarah attended a day centre where she had lots of friends. Since the pandemic she was unable to see them in person. Sarah reported that communicating digitally is ‘not the same’, and ‘my heart is breaking to see them again’.

Sarah was single and identified as a lesbian. She came out several years ago to her close friends and mother, all of whom were supportive of her sexuality. She longed to have a girlfriend and felt jealous of friends who were in relationships. Sarah felt lonely and created an imaginary girlfriend called Zara, who provided a degree of comfort.

### Physical health

Sarah had hearing problems which necessitated the use of hearing aids; however, she did not like to wear these due to experiencing recurring ear infections. Sarah struggled with weight management. Despite working hard at dieting, exercising, and completing healthy living courses, she was clinically obese with a BMI (kg/m^2^) around 36. She therefore experienced knee and back pain which a physiotherapy assessment reported was weight related. She also reported that due to her menstrual cycle she experienced pain, discomfort, dysregulated emotions, and food cravings for several days each month.

### Cognitive abilities

Sarah completed several cognitive assessments with her CLDN during a routine health check-up. The Adaptive Behavioural Assessment System Second Addition (ABAS-II) assesses the adaptive and social functioning of individuals who may have difficulties with daily skills [56]. This showed Sarah’s functioning to be in the fourth percentile, described as ‘borderline’. Her strengths were within the ‘social skills’ section. Sarah also recently completed the Dementia Questionnaire for people with learning disabilities (DLD) [26] which indicated no cognitive or functional decline.

### Risk

Sarah’s medical notes reported that she displayed ‘suicidal ideation’ by ‘collecting leaflets on suicide and passing these to staff’. The Royal College of Psychiatrists [67] reported that suicide is common amongst PWID, yet remains overlooked. Lunsky et al. [48] found higher prevalence rates amongst females and ‘high functioning individuals’, correlating with stress, anxiety, depression, and loneliness.

Whilst assessing the function of Sarah’s leaflet collection, it appeared that she likened comfort-eating to killing herself. She believed that comfort-eating would lead to physical health difficulties, such as diabetes, resulting in her premature death. The ‘suicidal ideation’ appeared to be associated with the loss of her grandfather, the loss of seeing her friends due to COVID-19, and her perception of the impact of comfort-eating on her long term health. The risk was reviewed and Sarah confirmed she had no immediate plans nor means to end her life. Protective factors included Sarah’s awareness of how sad her family would feel should she die like this, and the prospect of seeing her friends again. Discussions with the MDT confirmed no immediate risk to Sarah’s physical health in regards to her weight or comfort eating. Ongoing risk was managed via weekly discussions between the therapist, supervisor, Sarah and Gill.

## Case presentation: intervention

Remote working meant adapting the intervention and giving additional consideration to the therapeutic collaboration, therapeutic processes, systemic working, limitations, identifying and formulating the problem, applying change methods, and monitoring efficacy.

### Collaboration

In line with the manual of CBT for PWID and common mental health difficulties by Hassiotis et al. [32], the first stage of intervention focussed on collaboration between Sarah, Gill, and the therapist. This ensured co-working and that Sarah understood the goals and rationale of CBT. Evidence suggests that the quality of the client–therapist alliance is a reliable predictor of clinical outcomes, regardless of psychotherapeutic modality [4]. Lovett [49] supports this view further and stresses the importance of creating a collaborative therapeutic relationship within ID.

Sarah created rules and set her own goals which were adapted into a therapy contract. This was regularly referred to which helped build trust and rapport. The pace was deliberately slow and repetitive. Hassiotis et al. [32] reports this can enhance clients’ engagement and motivation. The therapist spoke slowly and interventions were deliberately repeated to encourage “overlearning”. Time was allocated for Sarah to provide feedback, and discuss whether the session had helped her to reach her goal of ‘no more comfort eating’. A midway review allowed Sarah to decide which therapeutic elements were helping her reach this goal, and which she would like to discontinue or change.

From the first session, Sarah shared her concern about how she would cope with the eventual ending of therapy. Sarah asked for sessions to ‘never end’, noting how she ‘hates saying goodbye’. The therapist proactively planned towards the end of therapy by asking Sarah to colour a pie chart (later renamed ‘pizza chart’, at Sarah’s request) segment after each session, symbolising the remaining sessions. This facilitated discussions around feelings, coping and planning for the last session: throwing a ‘party’ to celebrate Sarah’s progress.

### Systemic working

At Sarah’s request, Gill attended every session. Boundaries with Gill’s involvement were managed through continual checking-in and feedback at the end of each session. All interventions were designed with Gill’s input with the view that the inclusion of caregivers in therapy can enhance treatment effects through added encouragement and assistance with skills practice in natural contexts, outside sessions, and after treatment has ended [30].

The therapist regularly checked in with Gill as to how she was managing, as Dhiman et al. [23] notes the impact that COVID-19 can have on the mental health among caregivers of PWID. As significant changes can contribute towards adverse outcomes, they highlight the importance of a systemic approach in helping to safeguard caregivers. The systemic approach of integrating and consulting with Sarah’s mother helped to ensure improved engagement, motivational barriers were overcome, and any changes started in therapy are more likely to continue [74]. Additionally, Sarah consented to sharing formulations and letters with her GP, and including them on her medical notes, so that any professionals working with Sarah in the future can build on these.

### The therapeutic process

Sarah had 15 appointments in which she and the therapist each brought items for that session’s agenda. Sarah had the final decision on which topics time was allocated to. This meant, that even topics that at first seem unrelated to her goals were explored. For example, when Sarah opted to discuss her gemstones this initially seemed unrelated; however, the topic gave a better understanding of her spiritual beliefs, something which was later used as part of the intervention. This was in line with the person-centred approach recommended by Abbott and Howarth [1], incorporating clients’ unique interests, spiritual beliefs, and goals. After sessions, the therapist found images and words tentatively relating to cognitive and behavioural processes that had been discussed. These were posted to Sarah and Gill to facilitate meaningful discussion in the following session, subsequently using some in the formulation.

### Limitations

Remote working presented several challenges. One such challenge was communication difficulties. This was most prominent on the telephone, when Sarah declined to wear her hearing-aids, and before the therapist “tuned in” to Sarah’s communication style [32]. Thankfully, Gill helped with repeating, rewording, and providing context. Additionally, outcome assessments were not administered until halfway through the therapy due to the length of time required in establishing therapeutic rapport, goal setting, and identifying the problem.

Lastly, when reviewing the evidence base of PWID experiencing difficulties with comfort eating, there was a lack of specific references or resources; the majority of available literature revolved around dietetics and were not psychologically informed. Crawley’s [20] nutritional and practical guidelines for PWID were consulted, yet the source of difficulties appeared not to stem from a lack of knowledge about nutrition, rather from Sarah’s emotional and relational experiences.

### Identifying the problem

Sarah did not ‘overvalue of the importance of shape and weight and their control’, a key clinical feature of some eating disorders [54]. Neither did Sarah appear to be binge eating as she retained control during the episodes and was able to cease eating, before being struck by overwhelming feelings of guilt and disgust. These episodes were short, with quantities of food that did not appear extreme. Furthermore, Sarah did not report feeling uncomfortably full, or eating much more rapidly than normal. Assessment and supervisory discussions framed the comfort-eating as a potential response to managing difficult and intense emotions, rather than an eating disorder. This was conceptualised using a general CBT model, rather than exclusively focussing on difficulties around food, such as CBT-E.

Sarah used to receive additional care from her Mum and others around her feelings after comfort-eating. It was therefore hypothesised that by helping Sarah to create proactive strategies to manage and express her feelings, would reduce her cycle of comfort-eating, creating the basis of the formulation.

### Formulation

Multiple physical factors were considered, discussed with Sarah’s CLDN and eliminated as contributing towards her difficulties. These included side-effects from medication, natural menstrual cycle cravings, symptomatic relief from heartburn or acid-reflux and sensory processing difficulties. As a result, a psychological explanation formulated how comfort-eating met her emotional needs. Sarah experienced a huge amount of change, disruption, lack of control, and isolation through societal responses to COVID-19. Instead of seeking to simply alter Sarah’s cognitive and behavioural processes, it was important to keep in mind what may be possible for Sarah under the current circumstances, in line with her values and desires [36].

**Fig. 1:**
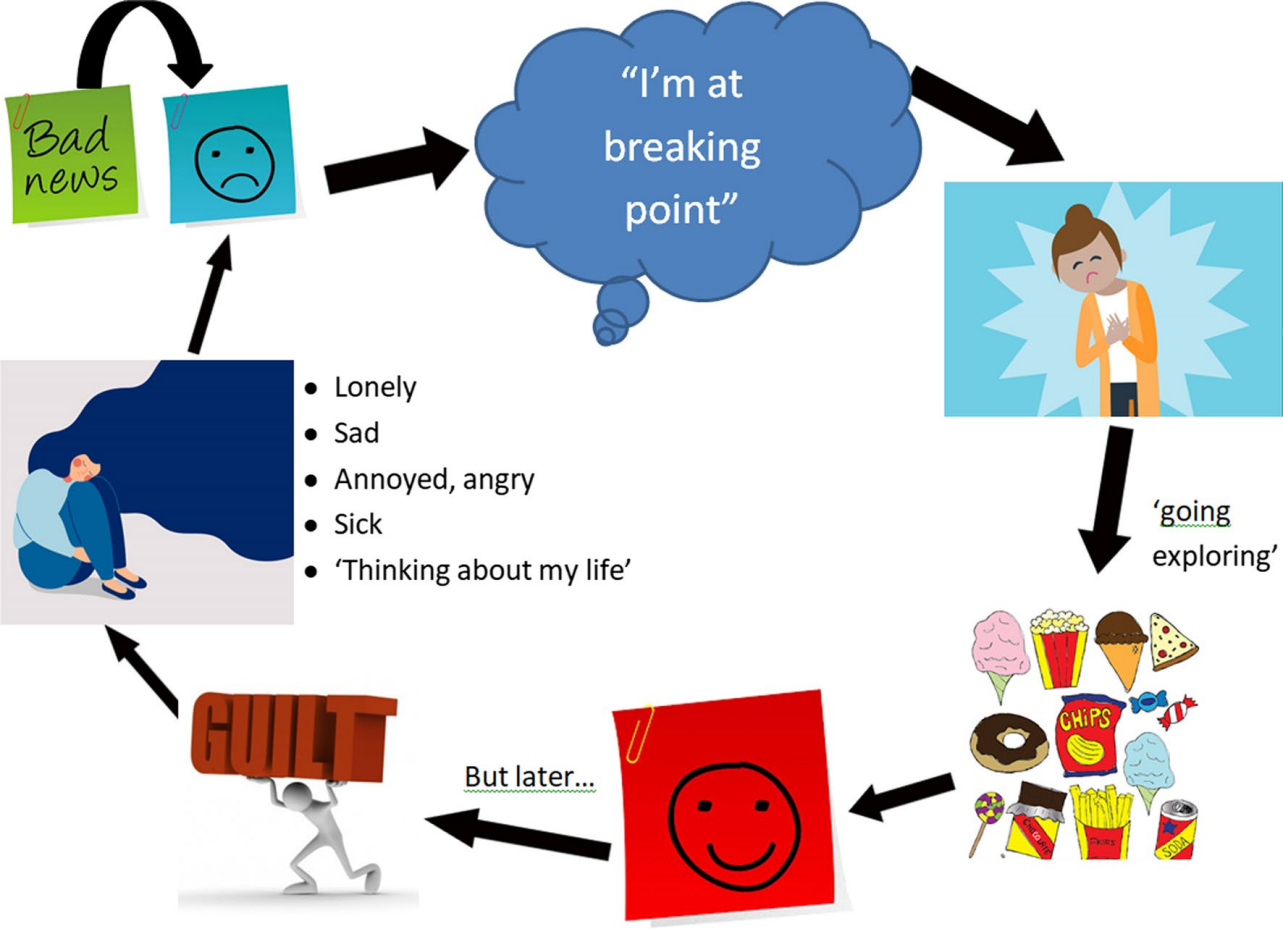
Maintenance cycle

A sketched individual case formulation was co-developed to make clear the links between stressors, emotions and eating behaviours (see Fig. 1). This focussed on Sarah’s emotional experience (sadness, boredom, etc.) towards her perceived lack of valued social role, along with her loneliness in being a single lesbian woman and the temporary gratification that comfort eating brought. This was followed by guilt, overthinking and frustration, which maintained a vicious cycle.

This maintenance model was selected as opposed to a longitudinal formulation [9] because Sarah’s difficulties had only existed for one year and she did not want to focus on the past.

The image of the girl sitting down represents the accompanying experiences listed by Sarah. These experiences or ‘bad news’ led to feeling unhappy and at ‘breaking point’. Sarah expressed this as her ‘heart breaking’, symbolised in the image. The thought of ‘going exploring’ then ensued in a bid to dispel this feeling, leading to comfort-eating. After feelings of happiness were succeeded by guilt, the cycle continued. The shared formulation enabled examination of intervening by altering cognitive and behavioural processes seemingly maintaining these difficulties.

### Application of change methods

Therapeutic approaches were person-centred by developing shared images (rainbow = Sarah/lesbianism), gestures (swiping up = avoiding), and pictures. This helped strengthen rapport and understanding, was in line with Sarah’s values [1], and demonstrated the therapist being gay affirmative [37]. Sarah took an active role in many cognitive behavioural therapeutic processes such as homework setting, even co-creating the diagrammatical formulation.

Firstly, Sarah identified that ‘bad news’ (regarding COVID-19) made her feel ‘terrible’ and was ‘everywhere’ (throughout the media). The therapist experimented in testing the impact of reducing exposure to these triggers on Sarah’s mood by helping her to monitor her exposure to them on TV, radio and at mealtimes. There appeared to be an immediate improvement because of this, as Sarah verbally reported enjoying ‘swiping it away’ initially on her phone, then jokingly as a gesture towards her surrounding environment, something which Gill also corroborates. Sarah also noticed that this made her feel less anxious and upset.

Next, Sarah opted to create a list of ‘coping strategies’ to manage her ‘heartache’, symbolised in Fig. 1. This list was expanded with activities that Sarah discovered she enjoyed and did not make her feel guilty afterwards. Sarah displayed this on her wall: referring to it when she was feeling sad and adding on new ideas. One of these strategies was ‘drawing how I feel’. The formulation had an image of someone (representing Sarah), sitting on the floor and ‘thinking about my life’, with many difficult emotions that Sarah regularly felt before and after comfort-eating. Instead of keeping these feelings inside, Sarah was encouraged and supported to process them through her artwork and accompanying stories. Sarah reported that this helped her ‘get things off my chest’.

Table 1 summarises how some of the main CBT interventions were adapted to work with a client with intellectual disability and via COVID-19 remote working.

**Table 1:** Therapy adaptations

Therapeutic strategies	Adaptations for intellectual disability and COVID-19
Engagement and rapport	Regular and predictable appointments, scheduled in advance Pictorial, easy-read therapy contract co-created with client and family Person-centred approaches e.g., repetition, use of language, shared agenda setting, gay affirmative therapy, etc All materials posted to the client and family in between sessions
Creating a shared case formulation	Maintenance cycles tentatively sketched, posted to client, then updated together in session Shared images and gestures used to co-create the formulation
Commencing self-monitoring	Remote mindfulness techniques in session, and practiced as homework Regular referral to the formulation, encouraging the client to recognise her current feelings and urges
Identification of high-risk situations	All instances of comfort eating and their antecedent events recorded, then discussed in session Basic behavioural experiments
Prevention of over-eating	Emotions underlying antecedent events targeted e.g., social strategies to prevent loneliness
Developing mood regulation skills	Client’s list of personal coping strategies to prevent comfort eating prominently displayed Encouragement of creative expression of emotions e.g., drawing
Enlisting help from others	Permission for family and MDT input sought from client
Relapse prevention	Continued practice of mood regulation skills Ongoing family support Formulation and intervention summary shared with family, Community Learning Disability Team, and day centre staff

### Outcome measures

Lambert et al. [43] suggest that feedback on clients’ therapy progress improves outcomes. The importance of outcome measures in clinical practice with PWID is recognised by the BPS Faculty for PWID [12]. Several measures monitored progress including three standardised measures, self-report data, frequency data, and clinical discussions. These helped the therapist understand whether Sarah’s needs were being met whilst building a picture of her mood, eating habits, and any maintaining factors.

The Maslow Assessment of Needs Scales-Learning Disabilities (MANS-LD) is a nineteen-item questionnaire for PWID to report how they feel that some of their basic needs are being met [64]. The BPS Faculty for PWID recommend using this to understand how service users perceive their quality of life [12]. The MANS-LD was used for two main reasons. Firstly, it was important to understand how Sarah’s difficulties with comfort-eating fitted with other aspects of her life, for example item number five: ‘how I spend my time’. Secondly, at the time of administering outcome measures there had already been several months of clinical work. As the MANS-LD can be applied retrospectively, it allowed assessment of whether there had been any change already, as opposed to measuring change from that time point onwards.

The Glasgow Depression Scale for people with a Learning Disability (GDS-LD) is a twenty-item tool that is reliable for screening and monitoring depressive symptoms amongst PWID [21]. Sarah completed this on session 7 of 15 scoring 23, and at the end scoring 13.

Additionally, Sarah completed The Glasgow Anxiety Scale for people with an Intellectual Disability (GAS‐ID). This is a twenty-seven-item psychometric evaluation scale which assesses state anxiety amongst PWID [53] and was completed on session 7 of 15 scoring 24, and at the end, scoring 16.

Throughout the intervention Sarah’s weight was not tracked. This was usually recorded by staff at the day centre; however, due to COVID-19 this had remained closed. Sarah opted not to keep a set of scales at home due to concerns that she would obsess over this.

As well as self-report data, frequency data was gathered verbally in sessions to monitor changes in Sarah’s comfort-eating. Sarah was guided to think of the moment immediately before incidents, so antecedent event information could be gathered. Table 2 shows a gradual reduction in instances of comfort-eating throughout therapy. Antecedent events provided evidence to support hypotheses of maintaining factors underpinning the formulation, whilst demonstrating that the interventions were working.

**Table 2:** Frequency of comfort eating

Date	Instances of comfort eating and antecedent events
November	
23rd–29th	No data recorded
30th–6^th^	2 (Antecedent events not recorded)
December	
7th–13th	2 (Antecedent events not recorded)
14th–20th	1 (Christmas dinner at day-centre, had to sit apart from others, ‘felt sad and lonely’
21st–27th	2 (‘Went exploring’ for ‘new and tasty food’ in house for Christmas)
28th–3rd	1 (‘Major episode’ as third lockdown announced)
January	
4th–10th	1 (‘Accidently ate Mum’s Christmas chocolates’, ‘felt terrible after’)
11th–17th	0
18th–24th	1 (Remembering ex-staff members who no longer sees, ‘felt sad and lonely’)
25th–31st	1 (‘On my period I crave comfort food but I don’t feel hungry’)
February	
1st–7th	0
8th–14th	1 (‘Felt sad about Valentine’s Day’, ate chocolate gifts intended for family)
15th–21st	0
22nd–28th	1 (‘Time of the month’, and friend suddenly died, ate care package from Grandma)
March	
1st–7th	0
8th–14th	0
14th–21st	0

Finally, the evaluation of clinical outcome was also considered in discussions with Sarah and Gill, who reported that problems with comfort-eating had subsided. Gill reported that previously ‘risky’ items from the supermarket including ice-lollies, biscuits, and multipacks of crisps would now remain untouched in the freezer and cupboards. Similarly, both Gill and Sarah reported that that Sarah’s mood was much improved, and she felt more self-confident.

### Discussion and conclusions

Sarah appeared to benefit from the remotely adapted CBT approaches previously described. Evidence supporting this was the reduction of target behaviours, improved outcome measures, and positive appraisals from Sarah and Gill. The adapted approach in self-monitoring the frequency of comfort eating (see Table 2) provided quantitative evidence to support the efficacy of the intervention. This method also highlighted the impact of antecedent events and their affective responses.

It became apparent throughout the work (and documented in the ABAS-II) that Sarah was highly able, independent and had good social skills. Sarah also had access to technology and was proficient at it. However, as Stott et al. [66] state, adapted-CBT is perhaps not suitable for all PWID and had Sarah not had this access or knowledge like other individuals, this may have posed difficulty.

Additionally, Sarah appeared motivated, and ready with a range of functional and social skills pertaining to talking therapy (e.g. holding a conversation [73]). Other PWID, however, may lack these abilities and require prior skills training, further adaptions, and increased ‘scaffolding’ [71].

Perhaps then, direct interventions adapted for PWID during COVID-19 may only be accessible to better supported, less disabled clients. Others may necessitate systemic approaches [50], thus the approach outlined in this case study may not be widely replicable. Rawlings et al. [58] also support this view, reporting that the majority of PWID were unable to participate in video therapy during COVID-19.

Another consideration is that therapy was conducted solely via telephone and video-conference. Whilst CBT techniques could be used via these mediums to alter unhelpful cognitive and behavioral patterns, this approach might not have allowed adequate consideration of the ever present, but invisible environmental factors [61, 65].

In considering gay affirmative therapy [37], there is a scarcity of information on how to adapt this for PWID [75]. Inequalities and a lack of progress may be mirrored in the scant evidence base of PWID who identify as lesbian, gay bisexual and transgender (LGBT). There are limited recent and consistent studies [24], most of which do not investigate sexuality from PWID’s perspective [61]. Indeed, Stoffelen et al. [65] stated that ‘very little is known regarding the personal experiences of lesbian… women with ID’. Within this case, focus was given to the need for a positive, accepting therapeutic relationship. In this application, it constituted a common factors approach to demonstrating cultural competence, understanding, and positive alliance. There was little available literature on how this should be adapted for work with PWID; the majority being either exploratory or descriptive [75]. This is despite the strong evidence that a strong therapeutic alliance is a reliable predictor of positive clinical outcome [4]. Given the disproportionate health disparities faced by PWID, one would expect that the unique needs and intersectionality of this population are of critical importance to further understanding.

This lack of clarity, coupled with some clients’ health needs being valued higher than their sexual needs [1], was felt as pressure and sadness by the therapist to understand Sarah’s unique experience from her perspective. Sarah’s longing to form an intimate relationship with another woman was evident in her creating an imaginary girlfriend: Zara. Additionally, lots of time was spent discussing Sarah’s sadness at being single, and unable to meet anyone in a romantic capacity. A social intervention may have been more beneficial, and certainly better received, than an individual mental health based intervention.

The author found no resources or guides for working with this problem, reflecting the scarcity of comfort-eating literature for PWID. This was despite it appearing to be fairly common through discussions with other clinicians. Similar to the lack of gay affirmative therapy information within ID, this may speak to bias towards and overlooking of PWID in healthcare policy and academic research [5].

Finally, emerging evidence suggests that COVID-19 lockdowns have led to widespread negative changes in eating such as more frequent eating, problems with control around food [60], and an increase in comfort food consumption [62]. Emotional-based comfort eating may therefore be a common reaction to stress, disrupted schedules and routines. Despite this, it was distressing for Sarah, leading her to treatment without pathologising or medicalising her experiences.

## Data Availability

All data and materials within this case study may be shared. Materials are available on request from the corresponding author.
